# Polycythemia Vera with Incidental Large Aortic Aneurysm and Giant Cell Arteritis: Case Report

**DOI:** 10.1177/11795476261445478

**Published:** 2026-05-04

**Authors:** Koutaibah Obaid, Khaled Sadek, Fatima Al Jaber, Mohammad Altermanini, Abdulrahman F. Al-Mashdali, Mohammed Yassin, Abdulaziz M. Alkhulaifi, Cornelia S. Carr, Shehab F. Mohamed

**Affiliations:** 1 Department of Internal Medicine, Hamad Medical Corporation, Doha, Qatar; 2 Department of Lab Medicine and Pathology, Hamad Medical Corporation, Doha, Qatar; 3 Department of Cardiology, Hamad Medical Corporation, Doha, Qatar; 4 Department of Hematology, National Center for Cancer Care and Research, Hamad Medical Corporation, Doha, Qatar; 5 Department of Cardiac Surgery, Hamad Medical Corporation, Doha, Qatar; 6 Department of Clinical Surgery, College of Medicine-Qatar University, Doha, Qatar

**Keywords:** polycythemia vera, giant cell arteritis, aortic aneurysm, case report

## Abstract

Polycythemia vera (PV) is a JAK2-mutated myeloproliferative neoplasm associated with thrombotic and vascular complications. Giant cell arteritis (GCA) is a granulomatous large-vessel vasculitis that may involve the aorta and can present as aortitis or aneurysm formation. Coexistence of PV and GCA is uncommon and may complicate diagnosis and management. We report a 41-year-old man with low-risk PV (JAK2 V617F–positive) who presented 4 months later with an ischemic stroke and was incidentally found to have a 5.0 cm ascending aortic aneurysm. Despite cytoreduction and surveillance, the aneurysm enlarged to 5.6 cm and was repaired electively. Histopathology of the resected aortic wall showed granulomatous inflammation with multinucleated giant cells and elastic lamina fragmentation, consistent with GCA. The patient had no cranial or systemic features suggestive of active GCA at diagnosis; therefore, systemic corticosteroids were deferred and close rheumatology follow-up was arranged. This case underscores the need to consider inflammatory aortitis/large-vessel vasculitis when atypical vascular findings occur in PV and highlights the value of multidisciplinary evaluation.

## Introduction

Polycythemia vera (PV) is a JAK2-mutated myeloproliferative disorder primarily marked by clonal erythrocytosis. Additional clinical manifestations include leukocytosis, thrombocytosis, splenomegaly, pruritus, constitutional symptoms, microcirculatory abnormalities, and an elevated risk of thrombosis. PV may also progress to myelofibrosis (post-PV MF) or acute myeloid leukemia (AML).

There are 2 primary classification systems for myeloproliferative neoplasms (MPN): the ICC3 and the WHO fifth edition. In relation to polycythemia vera (PV), the ICC criteria consist of 3 major and 1 minor criterion. The major criteria include: (i) hemoglobin/hematocrit (Hb/Hct) levels above 16.5 g/dL/49% in men and 16 g/dL/48% in women, or a red cell mass (RCM) more than 25% above the mean normal predicted value, (ii) a bone marrow biopsy revealing age-adjusted hypercellularity with trilineage proliferation (panmyelosis), characterized by prominent erythroid, granulocytic, and pleomorphic, mature megakaryocytes without atypia, and (iii) the presence of a JAK2V617F or JAK2 exon 12 mutation, with an assay sensitivity of <1%. The minor criterion is a subnormal serum erythropoietin (Epo) level. For an ICC diagnosis of PV, all 3 major criteria must be met, or the first 2 major criteria and the minor criterion must be satisfied. The WHO fifth edition criteria for PV are largely similar to the ICC guidelines but do not require RCM assessment.^1^

Giant cell arteritis (GCA) is a non-necrotizing granulomatous vasculitis that primarily affects large blood vessels and typically manifests in individuals over the age of 50. Despite extensive research, the exact triggers for the disease remain unidentified.^2^ The disease primarily impacts medium and large vessels, particularly the aorta and its branches, as well as cranial arteries. Giant cell arteritis (GCA) is frequently linked to musculoskeletal inflammatory conditions, such as polymyalgia rheumatica (PR).^3^

The coexistence of myeloproliferative neoplasms (MPNs) and autoimmune diseases has been extensively documented in the literature, particularly in patients with myelodysplastic syndrome and chronic myeloid leukemia (CML). Although the underlying mechanisms of this co-occurrence remain poorly understood, the association between these conditions is noteworthy. About 30 cases of co-incidence of GCA or other large vessel inflammatory conditions and myeloid neoplasms have been reported.^3^

Furthermore, several studies have discussed the association of JAK2V617F with aortic aneurysms and dissection. Experimental studies have shown that JAK2V617F mutation in bone marrow cells, mimicking human myeloproliferative neoplasms, accelerates atherosclerosis in hypercholesterolemic mice due to impaired red blood cell efferocytosis and inflammasome activation, and aggravates post-ischemic cardiac remodeling.^4^ One hypothesis is that JAK2V617F mutation induces a pathogenic inflammatory phenotype in adventitial tissue-resident macrophages, contributing to the development of aortic aneurysm and dissection.^4^

In this case report, we report an unusual case of JAK2 V617F–positive polycythemia vera complicated by stroke and a progressively enlarging ascending aortic aneurysm with incidental histopathologic findings consistent with giant cell arteritis, and to highlight key diagnostic considerations, differential diagnosis (including Takayasu arteritis), and management implications of large-vessel vasculitis in patients with myeloproliferative neoplasms.

## Case Presentation

A 41 years old previously healthy Indian gentleman was initially referred to the hematology clinic due to persistent elevation of hemoglobin after presenting with non-resolving headache and dizziness. Initial CBC showed WBC 12.0 × 10^3^/µL, RBC 9.7 × 10^6^/µL, hemoglobin 20.2 g/dL, hematocrit 64.6%, MCV 66.6 fL, and platelets 270 × 10^3^/µL. He denied systemic and cranial symptoms classically associated with giant cell arteritis, including new-onset headache, scalp tenderness, jaw claudication, visual symptoms (blurred vision, diplopia, transient, or permanent visual loss), constitutional symptoms (fever, unintentional weight loss, fatigue, night sweats), polymyalgia rheumatica symptoms (proximal shoulder/hip girdle pain and morning stiffness), limb claudication, and chest/back pain suggestive of active aortitis.

Vital signs on presentation were stable and he was afebrile (temperature 36.9, blood pressure 128/82, heart rate 84, respiratory rate 16, oxygen saturation 100% on room air). Physical examination revealed no temporal artery tenderness, nodularity, or reduced pulsation. Cardiovascular examination showed normal heart sounds without murmurs; peripheral pulses were palpable and symmetric with no audible bruits. Respiratory examination was unremarkable with clear breath sounds bilaterally. Abdominal examination was soft and non-tender with no organomegaly. Neurological examination was unremarkable; there were no visual deficits reported or observed at assessment.

His workup included peripheral smear, bone marrow biopsy with immunohistochemistry, cytogenetics, and molecular genetics. Peripheral smear showed hypochromic microcytic red blood cells with erythrocytosis and anisocytosis. Bone marrow biopsy was hypercellular with prominent panmyelosis and erythropoiesis. Cytogenetics analysis showed normal BCR/ABL1. However, molecular genetics analysis revealed positive JAK2 V617F missense mutation. As a result, the overall findings were consistent with myeloproliferative neoplasm fulfilling the diagnostic criteria for polycythemia vera. Subsequently, he was classified as low risk polycythemia vera, started on low dose aspirin and scheduled for regular phlebotomy sessions.

**Figure 1.**
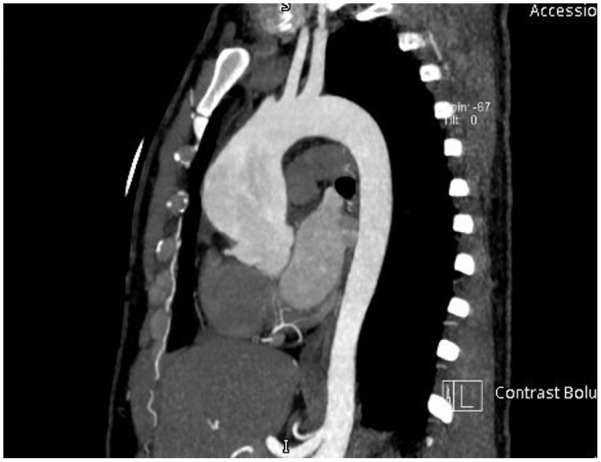
CT angiography of the thoracic aorta showing an ascending aortic aneurysm (maximum diameter approximately 5.0 cm) extending from the aortic root to the proximal arch.

**Figure 2.**
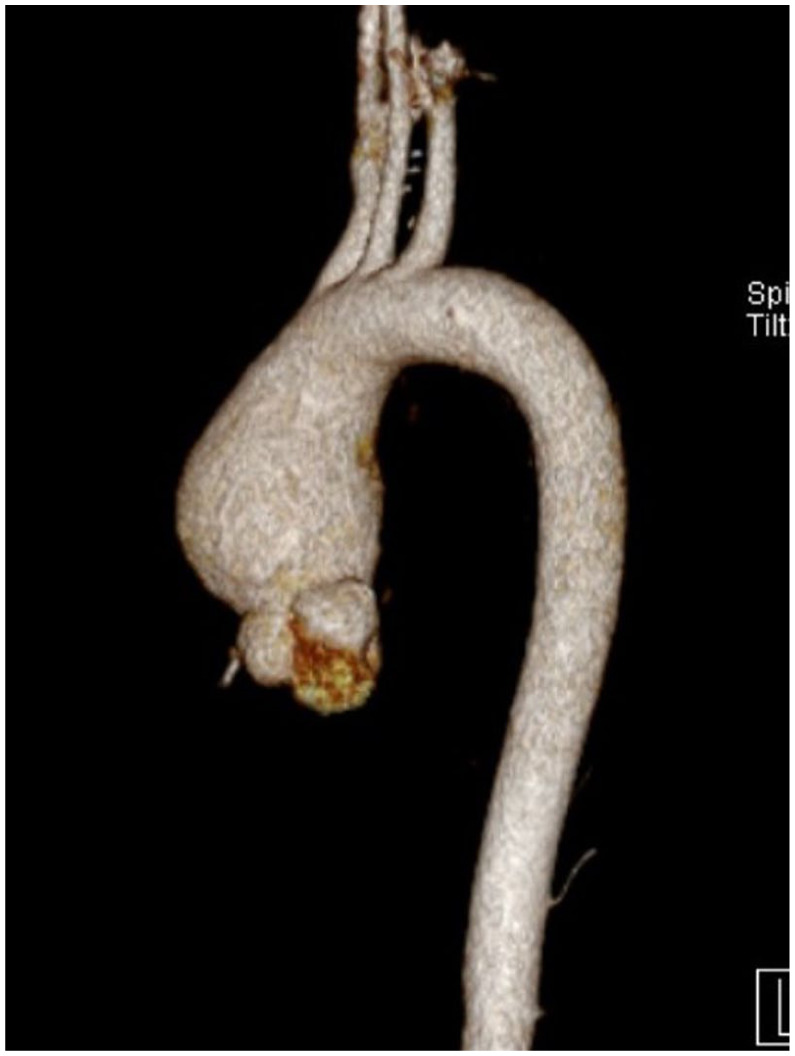
3D view of aortic aneurysm on initial CT scan.

Four months after diagnosis he presented to the hospital with imbalance and left upper limb weakness. Initial assessment revealed subcortical stroke, and he was admitted to an inpatient medical ward. During hospitalization MRI/MRA head confirmed watershed infarction and bilateral ICA stenosis more severe on the right side. As part of the stroke workup transthoracic echocardiography was obtained and revealed an incidental finding of a dilated ascending aorta measuring 4.9 cm in width. Thus, he was scheduled to undergo CT angiogram of the aorta which revealed aneurysmal dilatation of the ascending aorta measuring 5 cm in maximum diameter extending from the right to the level proximal to aortic arch (Figures 1 and 2). These findings were discussed with the cardiology team and advised outpatient follow-up in cardiology and cardiothoracic surgery clinic for further assessment and possible aneurysmal repair. After discussion with the hematology team the patient was started on hydroxyurea 500 mg twice daily and kept on aspirin 100 mg daily and high intensity statin for secondary prevention.

**Figure 3.**
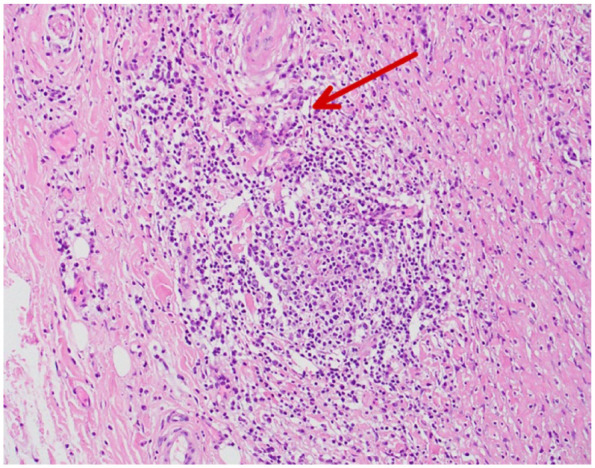
Histopathology of the resected ascending aortic wall showing chronic inflammatory infiltrates with multinucleated giant cells within the media, elastic lamina fragmentation and medial degeneration consistent with giant cell arteritis (indicated by the arrow).

**Figure 4.**
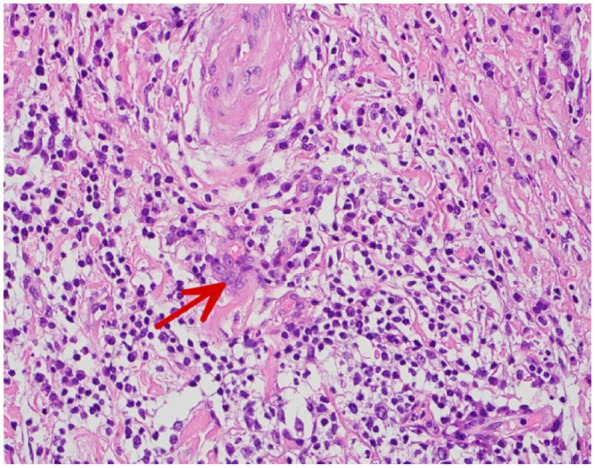
Histopathology of the resected ascending aortic wall showing chronic inflammatory infiltrates with multinucleated giant cells within the media, elastic lamina fragmentation and medial degeneration consistent with giant cell arteritis (indicated by the arrow).

Subsequently, follow-up CT scan after 18 months showed slight increase of the aneurysmal diameter to 5.6 cm in maximum diameter. Hydroxyurea was held as he was scheduled for elective repair of aortic aneurysm by interposition graft and normal hemoglobin level (14.2). The operation was successful without postoperative complications. An aortic aneurysm biopsy obtained during the operation revealed extensive chronic inflammation with a predominance of lymphocytes, plasma cells, and scattered eosinophils. Numerous multinucleated giant cells were present, often surrounding areas of medial degeneration and elastic fiber fragmentation. The inflammatory infiltrate extended through the media and into the adventitia, with associated fibrosis and disruption of the normal architecture (Figures 3 and 4). The biopsy result findings were consistent with the diagnosis of giant cell arteritis and the patient was referred to rheumatology clinic urgently for further assessment and management. During follow-up appointment in hematology clinic the patient reported he was compliant with hydroxyurea and aspirin. However, there were fertility concerns regarding hydroxyurea, thus, the medication had to be changed to interferon alfa. Inflammatory markers were reviewed at the time of rheumatology assessment; CRP was 2.6 mg/L, and ESR was not performed. Given the incidental histopathologic diagnosis after successful aneurysm repair and absence of cranial or systemic manifestations, systemic corticosteroids were not initiated, and close outpatient follow-up was arranged.

## Discussion

Polycythemia vera (PV) represents the most prevalent entity of myeloproliferative neoplasms (MPNs), which are associated with intricate symptom patterns that vary both within and across different MPN subtypes. Commonly observed symptoms in PV include insomnia, headaches, dizziness, vertigo, and depression among others. Suspected cases of PV require a comprehensive diagnostic workup that includes molecular evaluation.^5^ The WHO diagnostic criteria for PV incorporate molecular testing to detect JAK2 V617F or JAK2 exon 12 mutations. The JAK2 V617F mutation is the most predominant molecular aberration in PV, identified in more than 90% of affected individuals. A diagnosis of PV is established by fulfilling either all 3 major criteria or the first 2 major criteria in combination with the minor criterion.^6^

PV patients are at a heightened risk of thrombotic events, with arterial and venous thromboembolism being the primary contributors to morbidity and mortality in this patient population. Among PV patients, positive JAK2/V617F mutations, and previous thrombosis events are the main risk factors for stroke. The peak incidence of PV occurs between 50 and 70 years old, which is also a period of high-stroke incidence. Furthermore, PV may occur in persons of all age groups, including young adults and children, albeit rarely.^7^

Most thoracic aortic aneurysms (TAAs) are degenerative in nature and are commonly associated with atherosclerosis risk factors. Nonetheless, TAAs may also result from aortitis, which can develop in response to various systemic autoimmune disorders, including GCA.^8^ Aortic aneurysm (AA) has been identified as a potential complication in patients with myeloproliferative neoplasms (MPNs), in addition to other vascular complications. The JAK2 V617F mutation has been linked to a higher rate of vascular complications compared to other driver mutations. It has been suggested that JAK2 V617F may be a causal factor in the development of AA.^9^

The pathogenesis of GCA involves a breakdown in immune tolerance within the vascular wall, immune system aging, inflammation, and subsequent vascular injury and remodeling. The Janus kinase-signal transducer and activator of transcription (JAK-STAT) pathway is implicated in promoting inflammation in GCA in promoting inflammation in GCA. Diagnosing GCA requires a comprehensive clinical evaluation, supported by laboratory testing and vascular imaging. Histopathological examination of a vascular biopsy remains a critical step for confirming the diagnosis of GCA.^10^

Takayasu arteritis was considered in the differential diagnosis of large-vessel vasculitis; however, it was less likely given the lack of typical features such as limb claudication, reduced/unequal pulses, significant blood-pressure discrepancies, or characteristic imaging findings of long-segment stenosis/occlusion of the aorta and its major branches. In contrast, the histopathologic pattern of granulomatous inflammation with multinucleated giant cells and elastic lamina fragmentation supported GCA.^11^

Studies have investigated the association between autoimmune diseases and the development of MPNs. Research by Kristinsson et al found that individuals with a history of autoimmune disease had a 20% increased risk of developing an MPN. This risk was particularly pronounced in those with prior conditions such as giant cell arteritis, aplastic anemia, and Reiter’s syndrome.^12^ Conversely, a study by Elessa et al found no association between MPNs and autoimmune diseases. Their conclusion was based on observations that the prevalence of autoimmune diseases in their study cohort matched that of the general population.^13^ The activation of the JAK/STAT pathway, along with other mechanisms, has been proposed as a contributing factor to the observed association between myeloproliferative neoplasms (MPNs) and autoimmune diseases (ADs).^14^

The relationship between GCA and MPNs has been investigated in previous studies. A retrospective study of GCA cases compared 21 cases of GCA associated with MPNs to 41 controls with GCA but no associated MPNs. The findings indicated a stronger association of GCA with essential thrombocythemia compared to other MPN subtypes. Additionally, it was noted that patients with GCA and associated MPNs had shorter survival rates and exhibited fewer cephalic symptoms of GCA.^2^ In addition, a population-based study conducted in France revealed that individuals aged 50 years and above with giant cell arteritis (GCA) exhibited a higher incidence of myeloid hematological malignancies, particularly myeloproliferative neoplasms (MPNs) excluding chronic myeloid leukemia (CML).^15^ Furthermore, an analysis of the chronological sequence of diagnoses showed that a prior diagnosis of myeloid hematological malignancies, specifically MPNs other than CML, significantly increased the risk of developing GCA in men. However, this association was not evident in women. Based on this observation the authors hypothesized that clonal hematopoiesis might play a role in the development of GCA in some cases.^15^

The overlapping symptoms of MPNs and ADs pose a significant diagnostic challenge for clinicians. As highlighted previously, patients with MPNs may exhibit different presenting symptoms of a GCA compared to the general population. In this case, the initial presentation raised low suspicion for GCA due to the typical laboratory and molecular findings consistent with PV. Additionally, the presence of the JAK2 V617F mutation elevated the patient’s risk for complications such as stroke, aortic aneurysm, and GCA. Fortunately, the aortic aneurysm was incidentally identified, and further evaluation, including a biopsy, confirmed the diagnosis of GCA. This highlights the importance of a multidisciplinary approach in managing such complex cases to achieve the best possible outcomes. Given the not uncommon co-occurrence of MPNs with ADs and their overlapping symptom profiles, careful interpretation of clinical evaluations and laboratory investigations is crucial for accurate diagnosis and effective management.

Diagnostic limitations warrant emphasis. First, this patient’s age is atypical for classic cranial GCA, and the absence of cranial ischemic symptoms reduced clinical suspicion; large-vessel vasculitis may therefore be under-recognized when presenting primarily with aortitis/aneurysm. Second, inflammatory markers were limited (ESR not performed), and CRP was only mildly elevated, which may not exclude large-vessel vasculitis. Third, symptoms of myeloproliferative neoplasms can overlap with systemic inflammatory features, complicating bedside phenotyping. Finally, the diagnosis was made incidentally on surgical pathology; histopathology confirms vasculitis but cannot fully define disease extent or activity, underscoring the importance of longitudinal imaging and multidisciplinary follow-up.

In conclusion, patients with myeloproliferative neoplasms (MPNs) or autoimmune diseases (ADs) may have an increased risk of developing the other condition. Further research is essential to investigate the associations between specific subtypes of both entities. Clinicians should remain vigilant about this relationship when managing such cases. While shared mechanisms of pathogenesis, such as JAK/STAT pathway activation, have been proposed, additional studies are needed to deepen understanding. Moreover, exploring common therapeutic targets for these conditions could improve treatment strategies and outcomes for patients experiencing both MPNs and ADs.

## Patient Perspective

The patient reported initial confusion and concern upon receiving the diagnosis of polycythemia vera, a condition he had not previously encountered. Although his presenting symptoms—primarily headache and dizziness—were relatively manageable, the confirmation of a chronic myeloproliferative disorder heightened his awareness of potential complications. He expressed feeling reassured by the hematology team’s comprehensive explanation and was comfortable initiating low-dose aspirin and undergoing regular phlebotomy, which he found beneficial in the early phase of treatment.

Following the onset of neurological symptoms and subsequent hospitalization for an ischemic stroke, the patient described the experience as distressing, particularly given the unexpected diagnosis of a significant ascending aortic aneurysm. He reported appreciation for the multidisciplinary approach to his care, including initiation of hydroxyurea and appropriate secondary prevention strategies. After successful elective surgical repair of the aneurysm, he was informed that the histopathology was consistent with giant cell arteritis and was referred to rheumatology for further management. Despite the complexity of his clinical course, the patient conveyed gratitude for the coordinated and timely interventions that contributed to a positive outcome and ongoing disease management.

## Conclusion

This case illustrates a rare coexistence of polycythemia vera with an ascending aortic aneurysm due to histopathology-confirmed giant cell arteritis. In patients with myeloproliferative neoplasms who present with atypical vascular manifestations, clinicians should maintain a high index of suspicion for inflammatory aortitis and large-vessel vasculitis, even in younger individuals. Multidisciplinary evaluation, longitudinal imaging surveillance, and timely surgical and immunosuppressive management are essential to optimize outcomes.

## References

[1] Tefferi A Barbui T. Polycythemia vera: 2024 update on diagnosis, risk-stratification, and management. Am J Hematol. 2023;98(9):1465-1487. 37357958 10.1002/ajh.27002

[2] Papo M Friedrich C Delaval L , et al. Myeloproliferative neoplasms and clonal haematopoiesis in patients with giant cell arteritis: a case-control and exploratory study. Rheumatology. 2022;61(2):775-780. 33836046 10.1093/rheumatology/keab337

[3] Bogucka-Fedorczuk A Czyż A Szuba A , et al. Co-occurrence of unclassified myeloproliferative neoplasm and giant cell arteritis in a patient treated with allogeneic hematopoietic stem cell transplantation: a case report and literature review. Cent Eur J Immunol. 2021;46:121-126. 33897294 10.5114/ceji.2019.83140 PMC8056354

[4] Al-Rifai R Vandestienne M Lavillegrand JR , et al. JAK2V617F mutation drives vascular resident macrophages toward a pathogenic phenotype and promotes dissecting aortic aneurysm. Nat Commun. 2022;13(1):6592. 36329047 10.1038/s41467-022-34469-1 PMC9633755

[5] Gerds AT Gotlib J Ali H , et al. Myeloproliferative neoplasms, version 3.2022, NCCN clinical practice guidelines in oncology. J Natl Compr Canc Netw. 2022;20(9):1033-1062. 36075392 10.6004/jnccn.2022.0046

[6] Arber DA Orazi A Hasserjian R Thiele J Borowitz MJ Le Beau MM , et al. The 2016 revision to the World Health Organization classification of myeloid neoplasms and acute leukemia. Blood. 2016;127(20):2391-2405. 27069254 10.1182/blood-2016-03-643544

[7] Hui S Zhao J Huo T , et al. Ischemic stroke as an initial performance of polycythemia vera in young adults: a case report and literature review. Medicine. 2024;103(7):e36953. 10.1097/MD.0000000000036953 PMC10869076 38363912

[8] Ince H Nienaber CA. Etiology, pathogenesis and management of thoracic aortic aneurysm. Nat Clin Pract Cardiovasc Med. 2007;4(8):418-427. 17653114 10.1038/ncpcardio0937

[9] Yokokawa T Misaka T Kimishima Y , et al. Crucial role of hematopoietic JAK2 V617F in the development of aortic aneurysms. Haematologica. 2021;106(7):1910-1922. 33567809 10.3324/haematol.2020.264085 PMC8252954

[10] Pugh D Karabayas M Basu N , et al. Large-vessel vasculitis. Nat Rev Dis Primers. 2022;7(1):93. 34992251 10.1038/s41572-021-00327-5 PMC9115766

[11] Karabacak M Kaymaz-Tahra S Şahin S , et al. Childhood-onset versus adult-onset Takayasu arteritis: a study of 141 patients from Turkey. Semin Arthritis Rheum. 2021;51(1):192-197. 33383295 10.1016/j.semarthrit.2020.10.013

[12] Kristinsson SY Landgren O Samuelsson J Björkholm M Goldin LR. Autoimmunity and the risk of myeloproliferative neoplasms. Haematologica. 2010;95(7):1216-1220. 20053870 10.3324/haematol.2009.020412 PMC2895049

[13] Elessa D Zhao LP de Oliveira RD , et al. Clinical features and genomic landscape of myeloproliferative neoplasm (MPN) patients with autoimmune and inflammatory diseases (AID). Leukemia. 2023;37:1741-1744. 37433887 10.1038/s41375-023-01967-0

[14] Galimberti S Baldini C Baratè C , et al. Myeloid neoplasms and autoimmune diseases: markers of association. Clin Exp Rheumatol. 2022;40(1):49-55. 33427624 10.55563/clinexprheumatol/ddxmp9

[15] Greigert H Mounier M Arnould L , et al. Heamatological malignancies in giant cell arteritis: a French population-based study. Rheumatology. 2021;60(11):5408-5412. 33792672 10.1093/rheumatology/keab328

